# Overexpressing TGF-β1 in mesenchymal stem cells attenuates organ dysfunction during CLP-induced septic mice by reducing macrophage-driven inflammation

**DOI:** 10.1186/s13287-020-01894-2

**Published:** 2020-09-03

**Authors:** Feng Liu, Jianfeng Xie, Xiwen Zhang, Zongsheng Wu, Shi Zhang, Ming Xue, Jianxiao Chen, Yi Yang, Haibo Qiu

**Affiliations:** Department of Critical Care Medicine, Zhongda Hospital, School of Medicine, Southeast University, Nanjing, 210009 China

**Keywords:** Mesenchymal stem cells, Sepsis, Transforming growth factor-β1, Macrophage activation

## Abstract

**Background:**

Sepsis remains a leading cause of death in critically ill patients. It is well known that mesenchymal stem cells (MSCs) are a promising therapy partly due to their paracrine-mediated immunoregulatory function. Previous study demonstrated that transforming growth factor-beta1 (TGF-β1) is an important cytokine secreted by MSCs and that it participates in MSC-mediated macrophage phenotype switch from pro-inflammatory to pro-resolution. In addition, the transformation of macrophage phenotype may be a potential treatment for sepsis. However, the therapeutic effect of overexpressing TGF-β1 in MSCs (MSC-TGF-β1) on sepsis is not well understood. Therefore, this study aimed to evaluate the effects of TGF-β1 overexpressing MSCs on organ injury in cecal ligation and puncture (CLP)-induced septic mice and to detect the changes in macrophage phenotype during this process.

**Methods:**

Mouse MSCs stably transfected with TGF-β1 were constructed and injected into CLP-induced septic mice via tail vein. After 24 h, the mice were sacrificed; then, the histopathology of the organ was evaluated by hematoxylin-eosin (H&E) staining. Inflammatory cytokines were detected by ELISA. Macrophage infiltration and phenotype transformation in the tissues were determined by immunohistochemistry and flow cytometry. In addition, we performed adoptive transfer of mouse peritoneal macrophage pretreated with TGF-β1 overexpressing MSCs in septic mice.

**Results:**

We found that infusion of TGF-β1 overexpressing MSCs attenuated the histopathological impairment of the organ, decreased the pro-inflammatory cytokine levels and inhibited macrophage infiltration in tissues. TGF-β1 overexpressing MSCs induced macrophage phenotypes changed from pro-inflammatory to pro-resolution in inflammatory environment. The adoptive transfer of mouse peritoneal macrophages pretreated with TGF-β1 overexpressing MSCs also relieved organ damage in CLP-induced septic mice.

**Conclusion:**

Under septic conditions, TGF-β1 overexpressing MSCs can enhance the therapeutic effects of MSCs on organ injury and inflammation as a result of reduced macrophage infiltration and induced macrophages transformation, the adoptive transfer of macrophages treated with TGF-β1 overexpressing MSCs also relieved organ damage. This will provide new hope for the treatment of sepsis.

## Introduction

Sepsis is currently recognized as a life-threatening organ dysfunction caused by a dysregulated host response to infection that is associated with unacceptably high mortality [1–3]. Previous studies have observed that excessive inflammation results in organ damage [1, 4]. Multiple organ failure usually occurs in patients with sepsis and may increase the severity of sepsis and even lead to death [5]. The lungs are one of the most vulnerable target organs in sepsis, but sepsis also causes damage to other organs, such as the liver and spleen [6]. Studies have shown that for patients with sepsis, mortality rate significantly increased as the number of dysfunctional organ increased [7]. Furthermore, a prospective study found a significant increase in mortality in patients with shock compared to patients with sepsis, and the risk of death upon the involvement of additional affected organ increases by 15–20% [8].

Mesenchymal stem cells (MSCs) are multipotent stem cells that can be derived from bone marrow and many other tissues [9]. MSCs have previously been used in a septic mice model and have been shown to reduce hyper-inflammation and tissue damage while promoting the survival of these mice [10]. In addition, MSCs can secrete multiple paracrine factors to modulate the inflammatory response [11, 12]. MSCs can also be modified before systemic infusion to exert improved therapeutic effects. A previous study reported that IL-10-overexpressing MSCs exerted regulatory effects on the inflammatory response and improved the survival rate in a mouse model of endotoxin-induced acute lung injury [13]. Furthermore, studies have shown that in ARDS mice, the infusion of prostaglandin E2 overexpressing MSCs significantly inhibited the expression of the pro-inflammatory cytokine IL-1β and tumor necrosis factor-α in lung tissue, thereby improving lung injury in mice [14]. These results provide novel strategies for the clinical treatment of sepsis by suppressing the inflammatory response.

The physiology and pathology of organ dysfunction caused by sepsis are highly complex [15]. Evidence has suggested that macrophages are involved in the pathogenesis of severe sepsis [16]. In response to environmental stimuli, macrophages can be activated and polarized to different phenotypes and are involved in pro-inflammatory activities or inflammation resolution [17, 18]. Recent findings have shown that the imbalance between macrophage phenotype and consequent excessive inflammatory responses are related to sepsis progression. Thus, regulating the balance in macrophage phenotype in the early stages of sepsis is beneficial to the host. MSC-secreted immunosuppressive cytokines participate in regulating macrophage-mediated immune responses; for example, MSC-derived TGF-β modulates microglial activation [19].Our previous study showed that MSCs secretion of TGF-β1 induced LPS-stimulated macrophage phenotype switch from pro-inflammatory to pro-resolution [20]. In addition, clinical studies have also confirmed that for patients with sepsis, the plasma levels of TGF-β1 in surviving patients are significantly higher than those in death, the elevated TGF-β1 levels in sepsis is considered part of “compensatory anti-inflammatory response syndrome” [21, 22]. However, the therapeutic effects of TGF-β1 overexpressing MSCs (MSC-TGF-β1) on macrophage phenotypic transformation, as well as organ damage, in septic mice are not completely understood.

In this study, we established a mouse sepsis model with cecal ligation and puncture (CLP). To improve the immunomodulatory capacity and therapeutic potential of MSCs in sepsis, we constructed MSCs with high and stable TGF-β1 expression using a lentiviral vector. We first investigated the effects of MSC-TGF-β1 on sepsis-induced organ injury and inflammation. Second, we explored the effects of MSC-TGF-β1 on macrophage activation in septic mice. Furthermore, we used a co-culture system of MSCs and LPS-stimulated macrophages to observe the direct impact of MSC-TGF-β1 on macrophage activation in vitro. Finally, we performed adoptive transfer assays using macrophages pretreated with MSC-TGF-β1 in septic mice to determine their effects on tissue damage.

## Materials and methods

### Ethics statement

All studies used 6 to 8-week-old male wild-type (WT) C57BL/6 mice (Laboratory Animal Center, Yangzhou University, China), which were housed under standard conditions. All animal experiments in this study were approved by the Institutional Animal Care and Use Committee of Southeast University.

### Mesenchymal stem cell transfection and culture

MSCs derived from the bone marrow of C57BL/6 mice were purchased from Cyagen Bioscience, Inc. (Guangzhou, China). The MSCs were cultured in Dulbecco’s modified Eagle’s medium/F12 (DMEM/F12) containing 10% fetal bovine serum (Corning, Australia) in a humidified incubator with 5% CO_2_ at 37 °C. MSCs before passage six were used for in vivo TGF-β1 gene experiments. TGF-β1 gene overexpression was established using a lentiviral vector, and a green fluorescent protein (GFP)-overexpressing vector was used as an empty vector control. The lentivirus was packaged in 293T cells (Cyagen Biosciences, Inc.) with three packaging plasmids, and a higher titer of lentivirus was obtained. MSCs were transfected and selected using blasticidin. Subsequently, MSCs carrying GFP (MSC-normal control) or MSCs carrying both TGF-β1 and GFP (MSC-TGF-β1) were harvested.

### Quantitative real-time PCR analysis

Total RNA from MSCs, MSC-NC, and MSC-TGF-β1 was extracted using TRIzol reagent (Life Technologies, USA). RNA was reverse transcribed using Prime Script TM Trimester Mix (Takara, Japan) and subjected to RT-PCR with SYBR Premix Ex TaqTM11 (Takara, Japan) and a Step One Plus RT-PCR system (Life Technologies, USA). β-Actin was used as an endogenous control. RT-PCR primers were synthesized by Sangon Biotech (Shanghai, China) and the sequences were as follows: mouse β-actin forward primer, 5′-GGGAAATCGTGCGTGAC-3′, and reverse primer, 5′-AGGCTGGAAAAGAGCCT-3′, and mouse TGF-β1 forward primer, 5′-AGACGGAATACAGGGCTTTCGATTCA-3′, and reverse primer, 5′-CTTGGGCTTGCGACCCACGTAGTA-3′.

### CLP model of sepsis

The sepsis model was induced via the CLP method. Briefly, the mice were anesthetized with 5% chloral hydrate (400 mg/kg) by intraperitoneal injection, and their lower abdomen was then shaved. The mice were positioned on Styrofoam pads and disinfected with 75% ethanol. An incision was made along the abdominal midline to expose the caecum and avoid blood vessel damage. The cecum was tightly ligated 1 cm from the apex with a 3–0 silk suture and punctured with a 22-gage needle, and fecal material was pushed through the hole. Then, the abdominal incision was closed in two layers with 4–0 silk sutures. For the sham group, the caecum was exposed but not ligated or punctured. Immediately after surgery, percutaneous injection of 1 mL of pre-warmed (37 °C) normal saline was done into the recovering mice. Finally, gently place the mice’ back on a thermal blanket until mice recovers from anesthesia. Six hours after CLP, the tail veins of the mice were injected with MSC, gene-modified MSC, drugs, or PBS. To determine the effect of MSCs and MSC-TGF-β1 on CLP-induced sepsis, the mice were sacrificed 24 h after MSC, gene-modified MSC or drug injection, and the lungs, liver, spleen, and blood were collected for further analysis.

### Groups and drug administration

Mice were divided randomly into following groups: sham group, CLP group, MSCs group, MSC-NC (MSC-normal control, which means MSCs carrying GFP) group, MSC-TGF-β1 group, recombinant TGF-β1 group (rTGF-β1), TGF-β1 inhibitor group (SB), and TGF-β1 group+SB-431542 group (TGF-β1+SB). The tail veins of the mice were injected with 1 × 10^6^ MSCs or gene-modified MSCs in 0.2 mL of normal saline. As a control, mice were administered 0.2 mL of normal saline. For the rTGF-β1 group, mice were infused 1 μg recombinant TGF-β1 (PeproTech Inc., USA) through caudal vein before CLP. For the TGF-β1 inhibitor group, 0.5 mg SB-431542 was injected intraperitoneally at 30 min before CLP. For the TGF-β1+SB group, isometric TGF-β1 and SB-431542 was administrated, respectively.

### Body temperature

Use a rectal thermometer to measure the animal’s body temperature every 4 h within 24 h of sepsis induction.

### Histopathological analysis

Parts of the lungs, liver, and spleen of the mice were rapidly removed and fixed in 4% phosphate-buffered formaldehyde for 24 h. They were then dehydrated and embedded in paraffin and sectioned to a thickness of 4 μm. The sections were stained with hematoxylin-eosin (H&E). Pathological changes in the tissues were analyzed under a light microscope. Organ injury was analyzed blindly by a pathologist who viewed ten randomly selected fields in each section. The lung injury scores were calculated as previously described [22]. The liver injury scoring system was as follows: grade 0, no injury; grade 1, presence of degenerated hepatocytes with only rare necrosis foci; grade 2, small area of mild centrilobular necrosis around the central vein; grade 3, area of mild centrilobular necrosis more severe than grade 2; and grade 4, centrilobular necrosis more severe than grade 3; this scoring system has been described previously [23, 24]. The spleen injury scoring system was calculated according to Giamarellos-Bourboulis et al. [25]. The score for each tissue sample represents the mean score of ten different sections.

### Evaluation of the lung edema

Lung wet weight to body weight ratio (LWW/BW) was used for the assessment of pulmonary edema, which has described previously [26]. Briefly, the whole lung was removed and then cleared of all tissues outside the lung. Finally, the LWW/BW was calculated based on the values of lung wet weight and body weight (mg/g).

### Enzyme-linked immunosorbent assays

The levels of anti-inflammatory (IL-10) and pro-inflammatory (IL-6 and IL-1β) cytokines and TGF-β1 in the serum/cells were measured by enzyme-linked immunosorbent assay (ELISA) according to the manufacturer’s instructions (R&D Systems, American).

### Immunohistochemical staining

Lung, liver, and spleen sections were deparaffinized and hydrated. The slides underwent antigen retrieval at a high temperature and were blocked for 1 h using 5% goat serum (Sigma, D9663). After blocking, the sections were incubated overnight at 4 °C with primary antibodies (diluted 1:100). The primary antibodies used were against F4/80 (Servicebio: GB11027). After incubation, the sections were washed 3 times with PBS and incubated with a 1:50 dilution of biotinylated secondary antibody. The reaction products were incubated with diaminobenzidine (DAB, China) and then counterstained with hematoxylin. Positive areas were quantified with Image J. All images were captured under high-power magnification (× 400) using a light microscope (Olympus).

### Preparation of single-cell suspensions from mouse tissue

Lung tissue was tied off at the trachea and rinsed with PBS to remove erythrocytes. Ophthalmic scissors were used to gently cut the lung tissue into small pieces under aseptic conditions. Then, the samples were placed in a digestion solution containing collagenase V (Sigma, Germany) and incubated at 37 °C for 60 min. Then, the samples were placed on ice to terminate the digestion. The collected cell suspensions were filtered through nylon mesh, and treated with a Tris-NH4Cl RBC lysis solution (Beyotime, China). Mouse lung single-cell suspensions were obtained by adding 2 mL of PBS containing 1% BSA.

### Primary murine peritoneal macrophages and MSC-TGF-β1 co-cultures

Peritoneal macrophages were harvested from thioglycolate-injected C57BL/6 mice. The mice were injected intraperitoneally with 1.5 mL of 3% thioglycolate medium (Solarbio, China) for 3 days. Then, the ascites of the mice were collected and centrifuged, and the cells obtained were seeded in incomplete medium. After incubation in 5% CO_2_ at 37 °C for 3 h, the unattached cells were removed, and the adherent cells were cultured in complete medium. Peritoneal macrophages were stimulated with LPS (Sigma, Germany) at 500 ng/ml diluted in DMEM with 1% fetal bovine serum for 24 h. MSCs or MSC-TGF-β1 and macrophages were cultured at a ratio of 1:10 in a Transwell system.

### Macrophage phenotype analysis by flow cytometry

Macrophages were immunolabeled with antibodies against surface proteins. The following antibodies were used: anti-F4/80 BB700, anti-CD86 allophycocyanin (APC), and anti-CD206 phycoerythrin (PE) (BD Biosciences, San Diego, USA). Macrophages were collected, and an Fc receptor blocking agent was used to block Fc receptors (Miltenyi Biotech, Germany) for 5 min at 4 °C. Macrophages were incubated with antibodies in the dark at 4 °C for 30 min and then washed with PBS. The expression of macrophage markers was calculated based on the fluorescence intensity. All of the data were collected by flow cytometry using Novo Express (ACEA NovoCyte, China) and analyzed using Flow Jo software.

### Statistical analysis

Statistical analyses were performed using GraphPad Prism 7. For comparisons among multiple groups, we used one-way ANOVA followed by Tukey’s multicomparison post hoc test or Kruskal-Wallis *H* test, as appropriate. *P* < 0.05 was considered statistically significant.

## Results

### Lentiviral vector-mediated TGF-β1 expression in MSCs

**Fig. 1:**
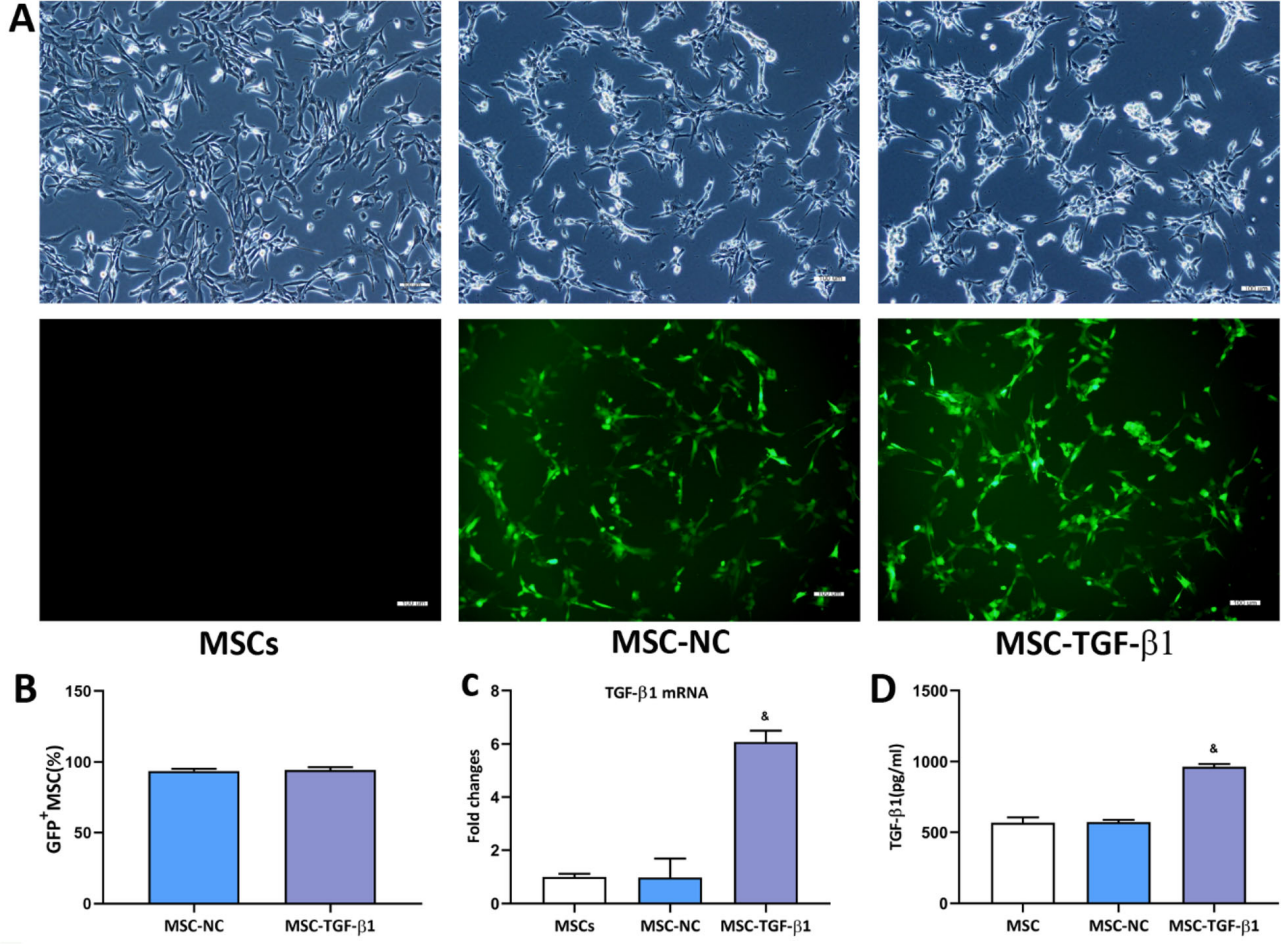
Measurement of TGF-β1 in genetically modified MSCs. **a** Green fluorescent protein was expressed in lentivirus-infected MSCs. Light microscopy (top) and fluorescence microscopy (bottom) images. Scale bar = 100 μm. **b** Quantitative analysis of lentivirus transduction. **c** TGF-β1 mRNA expression in MSCs, MSC-NC, and MSC-TGF-β1. **d** The expression of TGF-β1 in 2 × 10^6^ MSCs, MSC-NC, or MSC-TGF-β1 in 200 mL of culture supernatant, as measured by ELISA (*n* = 3; ^&^*p* < 0.05 vs. MSC-NC). The means ± SD of three experiments is shown. ELISA, enzyme-linked immunosorbent assay; MSCs, mesenchymal stem cells; MSC-NC, mesenchymal stem cell carrying GFP; MSC-TGF-β1, TGF-β1 overexpressing MSC

Firstly, we examined the therapeutic effects of rTGF-β1 on septic mice. Compared with the CLP group, the organ injury scores were significantly decreased in rTGF-β1 treatment group, while SB-431542 restrained the effects of rTGF-β1 on septic mice (Fig. S1). Then, we used lentiviruses to produce MSCs expressing genes encoding GFP and TGF-β1. Following transfection, MSCs expressed a green fluorescent signal (> 90%; Fig. 1a, b). Next, the TGF-β1 mRNA level in MSCs was detected by RT-PCR. The results showed that TGF-β1 mRNA expression was significantly increased in the MSC-TGF-β1 group compared with the MSC-NC group (Fig. 1c). The secretion of TGF-β1 in the MSC-TGF-β1 group was further detected by ELISA, and the concentration of TGF-β1 was obviously increased in the MSC-TGF-β1 group compared with the MSC-NC group (Fig. 1d).

### MSC-TGF-β1 attenuated sepsis-induced tissue injury

**Fig. 2:**
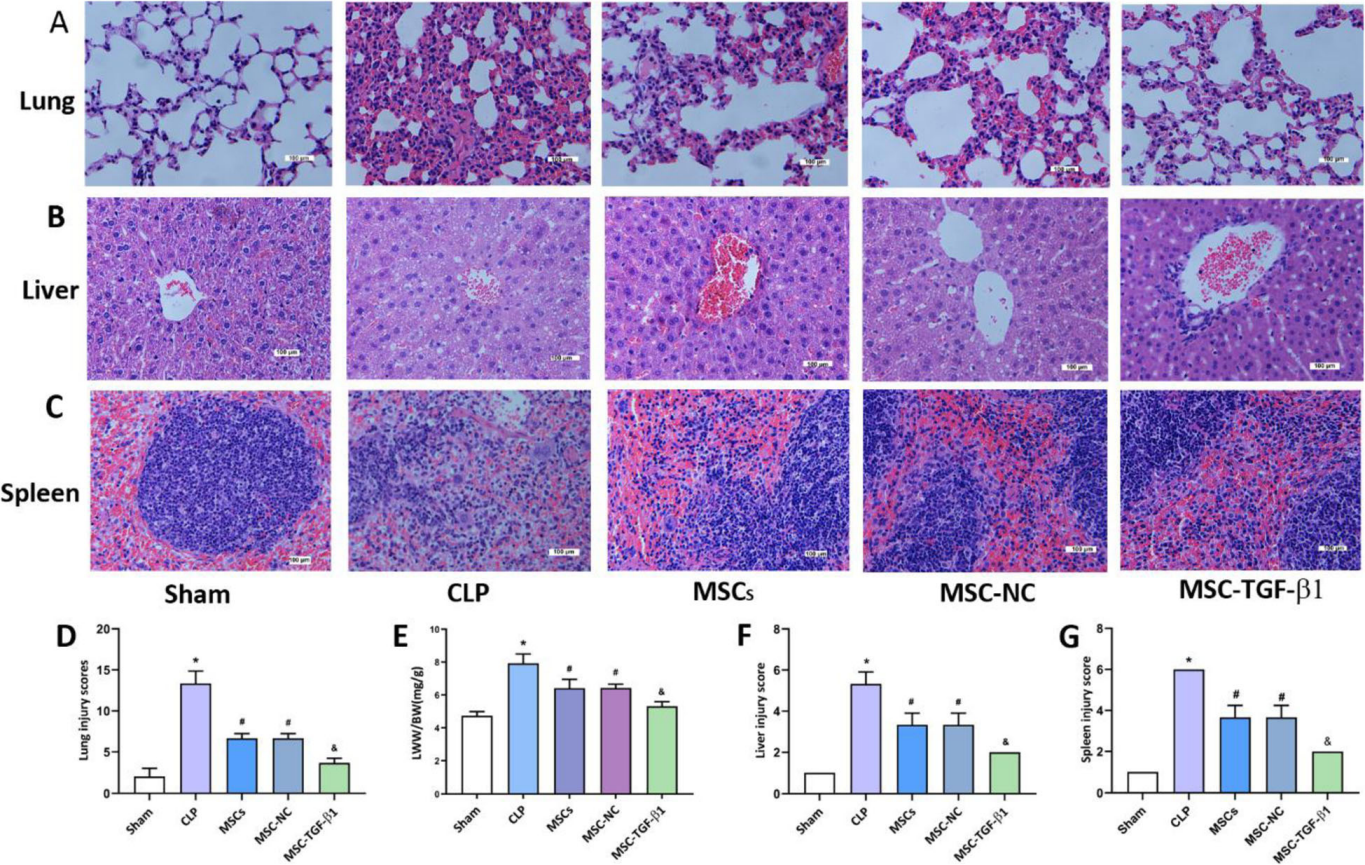
The effect of MSC-TGF-β1 on organ injury in CLP-induced septic mice. **a**–**c** Histopathological images of lung, liver, and spleen tissues (H&E staining, × 400). Scale bar = 100 μm. **d** The injury scores of the lung tissue. **e** Pulmonary capillary permeability was measured by the LWW/BW ratio. **f**, **g** The injury scores of the liver and spleen (*n* = 3; *p < 0.05 vs. sham group; ^#^*p* < 0.05 vs. CLP group; ^&^*p* < 0.05 vs. MSC-NC group). CLP, cecal ligation and puncture; MSCs, mesenchymal stem cells; MSC-NC, mesenchymal stem cell carrying GFP; MSC-TGF-β1, TGF-β1 overexpressing MSC

To investigate whether MSC-TGF-β1 had protective effects against CLP-induced sepsis in mice, MSCs, MSC-NC, or MSC-TGF-β1 were injected at 6 h after CLP induced sepsis; the mice were sacrificed at 24 h after MSC injection. Considering that the treatment of patients with sepsis relies heavily on the monitoring of vital signs, and body temperature is one of the important indicators [27], we firstly monitored the changes in body temperature during the treatment of MSC through a rectal probe every 4 h. During the 24-h study period, the body temperature of CLP mice was significantly lower than that of sham mice, after treatment with MSCs; the body temperature of the mice can be slightly increased. In addition, there was no significant difference in body temperature between MSCs and MSC-TGF-B groups (Fig. S2). Secondly, the histology was determined by H&E staining in lung, liver, and spleen sections from the mice. Compared with the findings of the sham group, the pathological features of the lungs in the CLP group were as follows: alveolar wall thickening, alveolar congestion, hemorrhage, and edema (Fig. 2a). In addition, the lung injury scores of the CLP group mice were markedly higher than those of the sham group (Fig. 2d). However, compared to CLP alone, the administration of MSCs and MSC-NC alleviated the histopathologic characteristics and lung injury scores (Fig. 2a, d). The effect was stronger in the MSC-TGF-β1 group than in the MSC-NC group (Fig. 2a, d). Furthermore, we calculated the lung LWW/BW ratio of each group to evaluate lung edema. The LWW/BW ratio was markedly increased in the CLP group compared with the sham group (Fig. 2e). After the administration of MSCs and MSC-NC, the LWW/BW ratio was significantly reduced, and the LWW/BW ratio was decreased significantly in the MSC-TGF-β1 group (Fig. 2e). The tissue sections were examined by fluorescence microscopy, GFP-labeled MSC can be observed in the lung of septic mice (Fig. S3A).

As shown in Fig. 2b, the livers in the CLP mice exhibited hepatocyte swelling and necrosis. Meanwhile, in the spleen sections from CLP mice, the corticomedullary border was blurred, white pulp and red pulp were loosely distributed, the spleen nodules were loosely distributed, and the lymphocytes were disorderly distributed (Fig. 2c). Meanwhile, the injury scores of the liver and spleen of CLP mice were markedly higher than those of sham mice (Fig. 2f, g). MSCs or MSC-NC injection relieved the severity of liver and spleen injury and the tissue damage scores were reduced. The protective effect on histology was considerably enhanced in the MSC-TGF-β1 group compared to the MSC-NC group (Fig. 2b, c, f, g). The tissue sections were examined by fluorescence microscopy; GFP-labeled MSCs can be observed in the liver and spleen of septic mice (Fig. S3B-C).

These findings show that TGF-β1 overexpression can effectively reduce tissue damage in septic mice.

### MSC-TGF-β1 reduced the inflammatory response by decreasing macrophage infiltration in organ

**Fig. 3:**
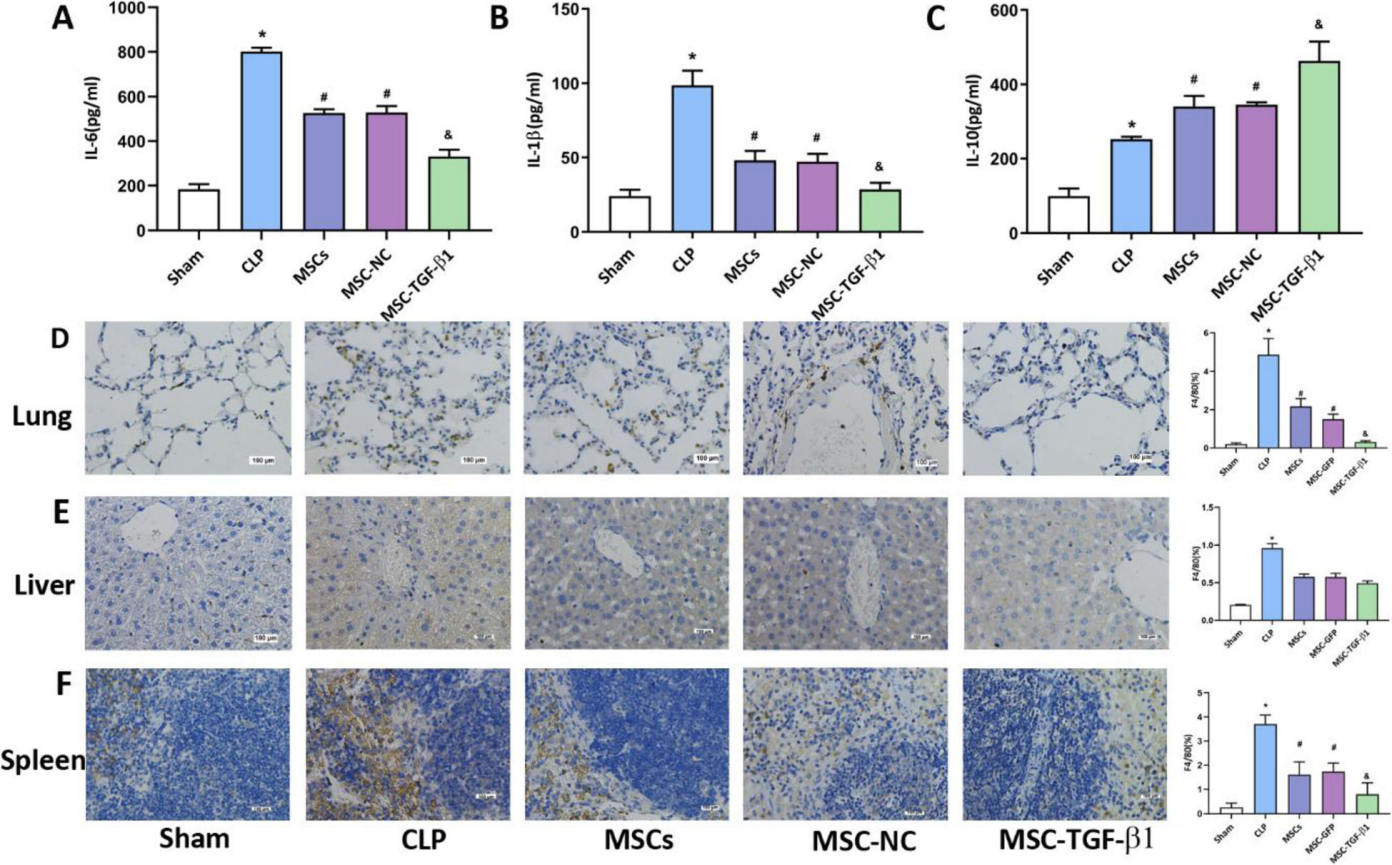
Effect of MSC-TGF-β1 on inflammatory cytokines in the plasma and macrophages in tissue. **a**–**c** The levels of IL-6, IL-1β, and IL-10 in the plasma were tested by ELISA (*n* = 3, *p < 0.05 vs. the sham group; ^#^*p* < 0.05 vs. the CLP group; ^&^*p* < 0.05 vs. the MSC-NC group). **d**–**f** The levels of macrophages in lung, liver, and spleen tissue were assayed by immumohistochemical staining (*n* = 3, *p < 0.05 vs. the sham group; ^#^*p* < 0.05 vs. the CLP group; ^&^*p* < 0.05 vs. the MSC-NC group). Scale bar = 100 μm. CLP, cecal ligation and puncture; MSCs, mesenchymal stem cells; MSC-NC, mesenchymal stem cell carrying GFP; MSC-TGF-β1, TGF-β1 overexpressing MSC

To assess the level of systemic inflammation, the levels of plasma inflammatory cytokines in each group were assessed by ELISA. Compared with that in the sham group, the concentration of pro-inflammatory cytokines, such as IL-6 and IL-1β, was markedly increased in the CLP group (Fig. 3a, b). IL-10 levels were also increased in the CLP group versus the sham group (Fig. 3c). Compared with CLP alone, MSCs or MSC-NC injection reduced the levels of IL-6 and IL-1β and enhanced the expression of IL-10 (Fig. 3a–c). Moreover, MSC-TGF-β1 treatment more significantly reduced the expression of IL-6 and IL-1β and enhanced the levels of IL-10 (Fig. 3a–c). Furthermore, we examined the expression of TGF-β1 in each group, compared with that in the sham group, the concentration of TGF-β1 was markedly increased in the CLP group, MSCs or MSC-NC injection enhanced the expression of TGF-β1, the level of TGF-β1 was more significantly increased in MSC-TGF-β1 treatment group (Fig. S4). The macrophages in our study were considered to be a heterogeneous population. They may be formed by the combination of resident macrophages and infiltrating monocytes that quickly acquire macrophage markers. F4/80 is a less specific marker as they can be expressed by macrophages and cells with monocytic origin. To assess macrophage infiltration in tissue, lung, liver and spleen sections were assessed for the expression of F4/80 by immunohistochemistry; we used the proportion of F4/80 to mean the F4/80 positive cells to the total cells. The results revealed that the number of F4/80-positive macrophages was obviously increased in the CLP group, while treatment with MSCs or MSC-NC reduced the number of F4/80-positive macrophages in the CLP-induced septic mice. Moreover, the number of F4/80-positive macrophages was markedly decreased in the MSC-TGF-β1 group compared to the MSC-NC group (Fig. 3d). In the livers and spleens of septic mice, MSCs, MSC-NC, and MSC-TGF-β1 had similar therapeutic effects, as described above (Fig. 3e, f). The above results verified that MSC-TGF-β1 can reduce systemic inflammation and reduce macrophages in the organ of CLP-induced septic mice.

### Effects of MSC-TGF-β1 on macrophage phenotype in septic mice

**Fig. 4:**
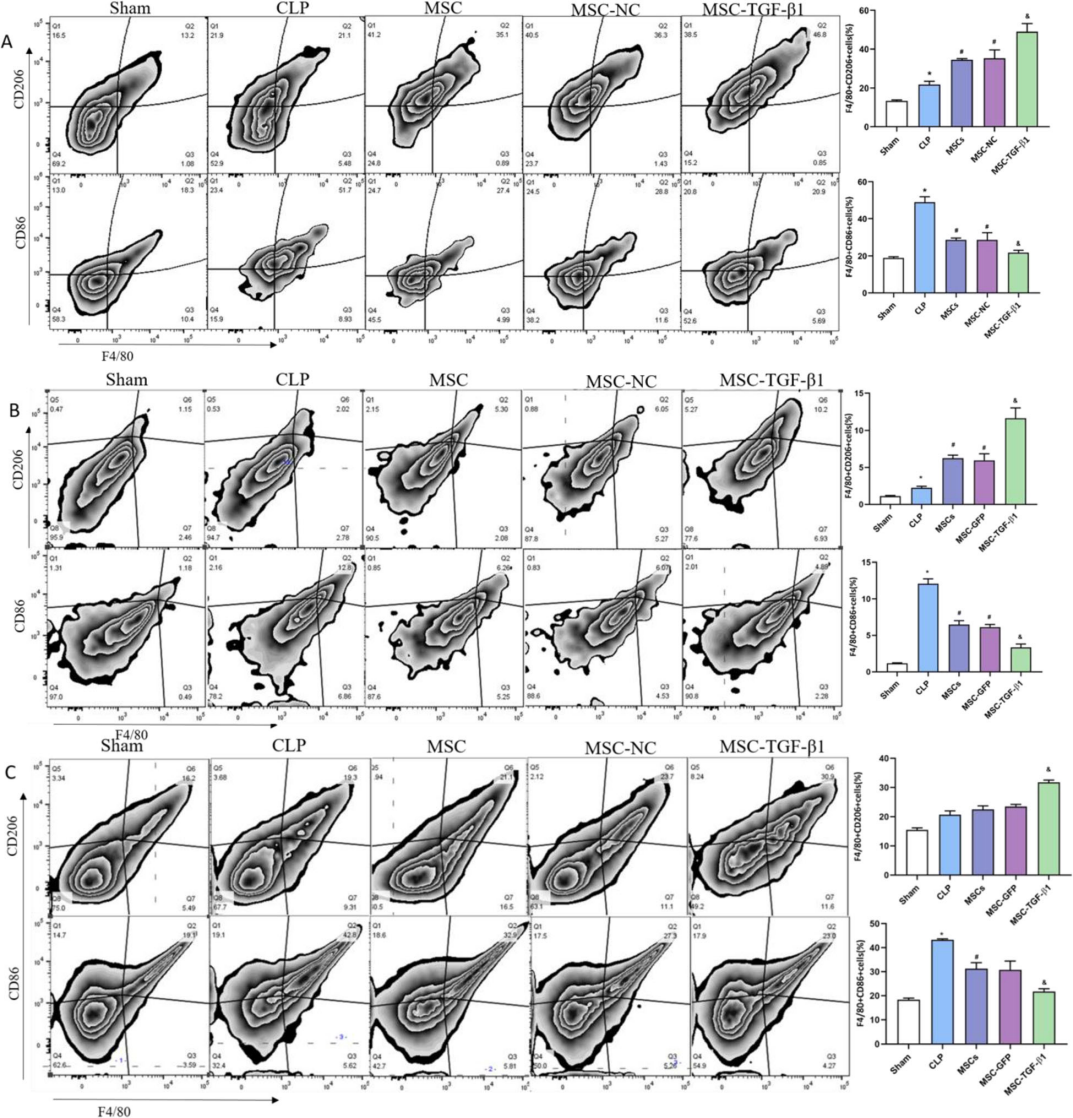
Effect of MSC-TGF-β1 on macrophage phenotype in septic mice. **a** The expression of CD86^+^ macrophages and CD206^+^ macrophages in the lung of each group was detected by flow cytometry. **b** The expression of CD86^+^ macrophages and CD206^+^ macrophages in the liver of each group was detected by flow cytometry. **c** The expression of CD86^+^ macrophages and CD206^+^macrophages in the liver of each group was detected by flow cytometry (*n* = 3, *p < 0.05 vs. the sham group; ^#^*p* < 0.05 vs. the CLP group; ^&^*p* < 0.05 vs. the MSC-NC group). CLP, cecal ligation and puncture; MSC, mesenchymal stem cell; MSC-NC, mesenchymal stem cell carrying GFP; MSC-TGF-β1, TGF-β1 overexpressing MSC

To determine the effect of MSC-TGF-β1 on macrophage phenotype, flow cytometry was performed. We assessed the expression of CD86 as a surrogate marker of macrophage pro-inflammatory activity, whereas CD206 was used as a marker of a pro-resolution phenotype. F4/80^+^ cell were first gated as macrophages and then categorized as CD86^+^ and CD206^+^ macrophages based on the expression of CD86 and CD206. Our results revealed that MSCs or MSC-NC treatment markedly reduced the ratio of CD86^+^ cells and increased the ratio of CD206^+^ cells in the lung of septic mice (Fig. 4a, b). However, compared to that in the MSC-NC treatment group, a significantly greater ratio of CD206^+^ cells was observed in the MSC-TGF-β1 group, while the ratio of CD86^+^ cells was decreased (Fig. 4a). In the livers and spleens of septic mice, results similar to those described above were found (Fig. 4b, c). The above results illustrated that MSC-TGF-β1 can induce the CD86^+^ cells to the CD206^+^ cells phenotype in the organ of CLP-induced septic mice.

### MSC-TGF-β1-induced LPS-stimulated macrophages polarized and reduced inflammation in vitro

**Fig. 5:**
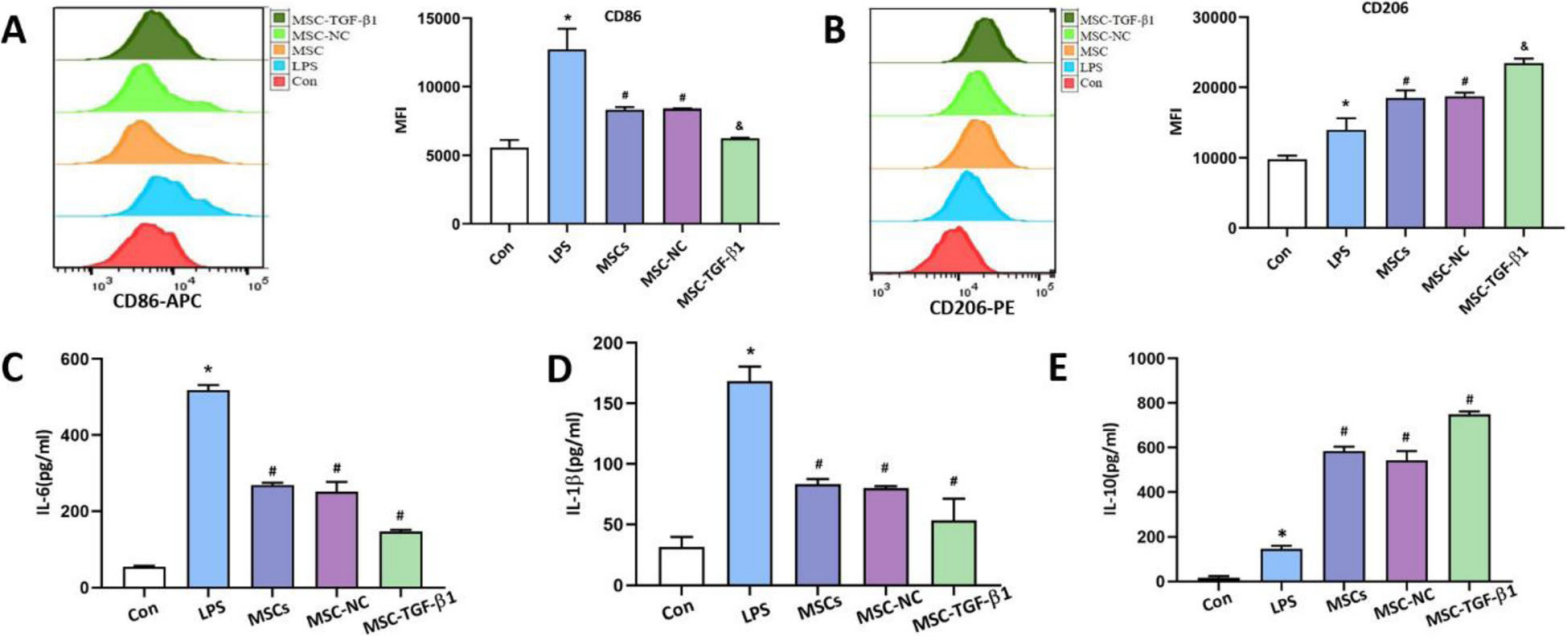
Effect of MSC-TGF-β1 on the phenotype of LPS-stimulated primary peritoneal macrophages. **a**, **b** The MFI of CD86 and CD206 in each group were detected by flow cytometry (*n* = 3, *p < 0.05 vs. the sham group; ^#^*p* < 0.05 vs. the CLP group; ^&^*p* < 0.05 vs. the MSC-NC group). **c**–**e** The concentrations of IL-6, IL-1β, and IL-10 in macrophages were tested by ELISA (*n* = 3, *p < 0.05 vs. the sham group; ^#^*p* < 0.05 vs. the CLP group; ^&^*p* < 0.05 vs. the MSC-NC group). ELISA, enzyme-linked immunosorbent assay; MFI, mean fluorescence intensity; MSCs, mesenchymal stem cells; MSC-NC, mesenchymal stem cell carrying GFP; MSC-TGF-β1, TGF-β1 overexpressing MSC

Based on these in vivo findings, we next examined the effects of MSC-TGF-β1 on macrophages in vitro. We explored the expression of CD206 and CD86 in macrophages by flow cytometry. Our results showed that LPS significantly increased the expression of CD86 and slightly increased the expression of CD206 (Fig. 5a, b). However, after stimulation by LPS, macrophages co-cultured with MSCs or MSC-NC showed lower expression of CD86 and higher expression of CD206 (Fig. 5a, b). Furthermore, treatment with MSC-TGF-β1 significantly increased the expression of the CD206^+^ marker and decreased the expression of the CD86^+^ marker (Fig. 5a, b). We further tested the levels of IL-6, IL-1β, and IL-10 in macrophages by ELISA. First, we detected the expression of inflammatory cytokines in MSC conditioned medium (Fig. S5), and then we collected the cell supernatant in the co-culture system; our results showed that the levels of IL-6 and IL-1β were obviously increased in the LPS-treated group (Fig. 5c–e). However, after co-culture with MSC or MSC-NC, a significantly greater level of IL-10 was observed, while the expression of IL-6 and IL-1β was decreased; the above changes were more obvious in the MSC-TGF-β1 group (Fig. 5c–e). These results indicated that MSC-TGF-β1 mediated the activation of LPS-stimulated macrophages and reduced excessive inflammation.

### Adoptive transfer of murine macrophages treated with MSC-TGF-β1 conferred protection against CLP-induced organ injury

**Fig. 6:**
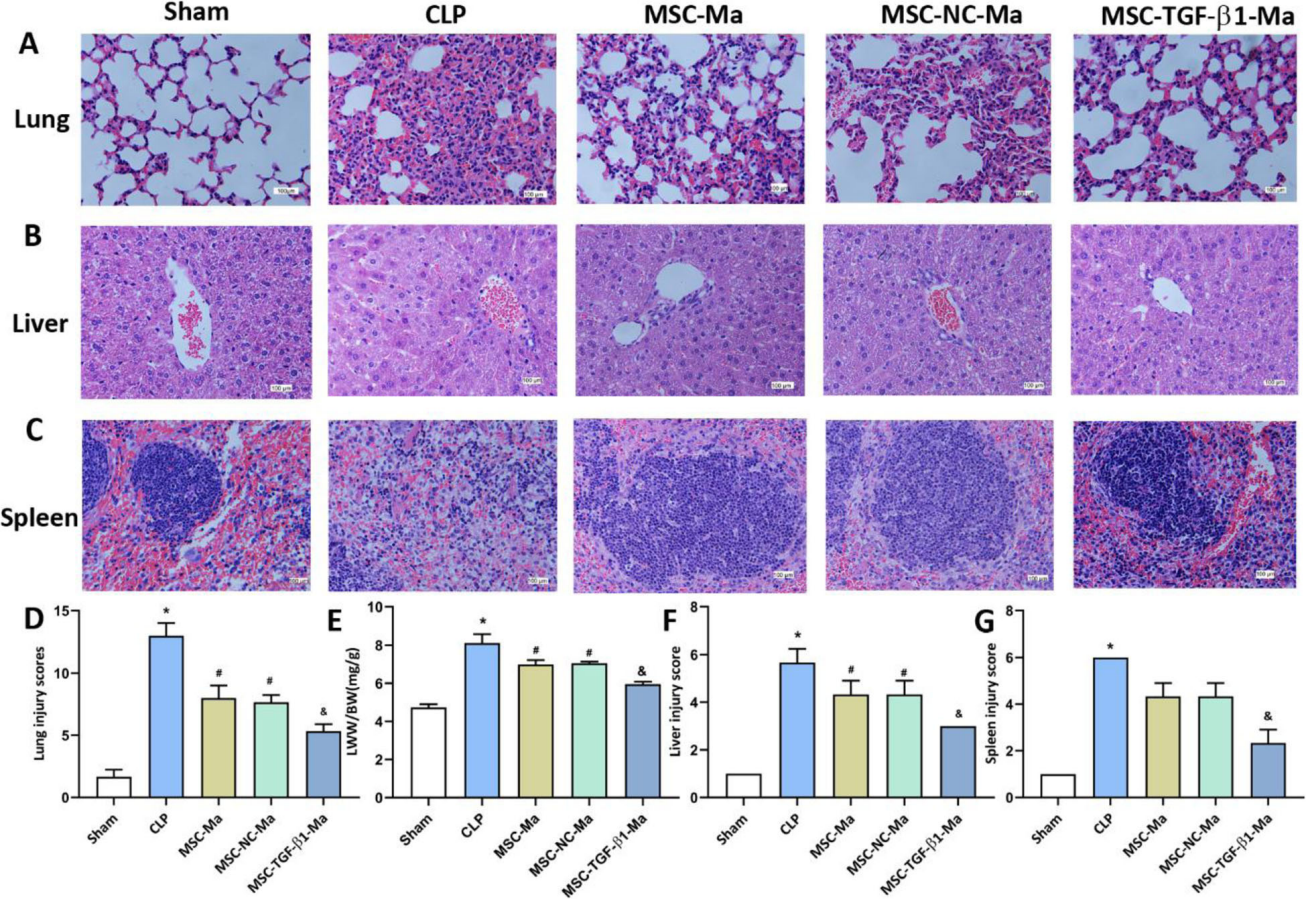
Effect of primary peritoneal macrophages treated with MSC-TGF-β1 on organ injury in CLP-induced septic mice. **a**–**c** Histopathological images of lung, liver, and spleen tissues were obtained by H&E (× 400). Scale bar = 100 μm. **d**–**g** Injury scores for the lung, liver, and spleen from each group (*n* = 3, *p < 0.05 vs. the sham group; ^#^*p* < 0.05 vs. the LPS group). **e** Pulmonary capillary permeability was measured by the LWW/BW ratio (*n* = 3, *p < 0.05 vs. the sham group; ^#^*p* < 0.05 vs. the LPS group). Ma, macrophages; MSCs, mesenchymal stem cells; MSC-Ma, MSCs preconditioned macrophage; MSC-NC-Ma, MSC-NC preconditioned macrophage; MSC-TGF-β-Ma, MSC-TGF-β preconditioned macrophage; MSC-NC, mesenchymal stem cell carrying GFP; MSC-TGF-β1, TGF-β1 overexpressing MSC

To detect whether the modulation of macrophages by MSC-TGF-β1 has a therapeutic effect in vivo, mouse peritoneal macrophages were isolated from C57BL/6 mice. We used LPS to induce macrophages activation, characterized by high CD86 expression, and then treated them ex vivo with MSCs or MSC-TGF-β1 for 48 h to induce phenotype transformation. Then, the MSC-TGF-β1-induced macrophages (1 × 10^6^) were adoptively transferred through the tail vein to mice that were challenged with CLP to induce sepsis. Compared with that in the CLP group, the pathological damage to the lung tissues of the group injected with peritoneal macrophages treated with MSCs or MSC-NC was reduced (Fig. 6a), and the lung injury scores and LWW/BW ratio were also lower than those in the CLP group (Fig. 6d, e). In addition, after the administration of peritoneal macrophages treated with MSCs or MSC-NC, pathological damage to the liver and spleen tissues were also improved (Fig. 6b, c). Furthermore, compared to that in the MSC-NC treatment group, pathological damage to the lung tissues was significantly improved, the tissue damage scores and LWW/BW ratio were decreased in the MSC-TGF-β1 group (Fig. 6a, d, e). The pathological damage and organ injury scores results in the liver and spleen were similar (Fig. 6b, c, f, g). Furthermore, administration of MSC-TGF-β1 pretreated macrophage attenuated the lung injury 7 days after transfusion via tail vein (Fig. S6). These findings demonstrate that the adoptive transfer of MSC-TGF-β1 induced macrophages in septic mice can relieve organ injury. More importantly, MSCs or MSC-TGF-β1-pretreated macrophage infusion not only had a short-term effect on septic mice, but also had a significant effect even after 7 days.

### Macrophage depletion weakened the therapeutic effect of MSC-TGF-β1 in septic mice

**Fig. 7:**
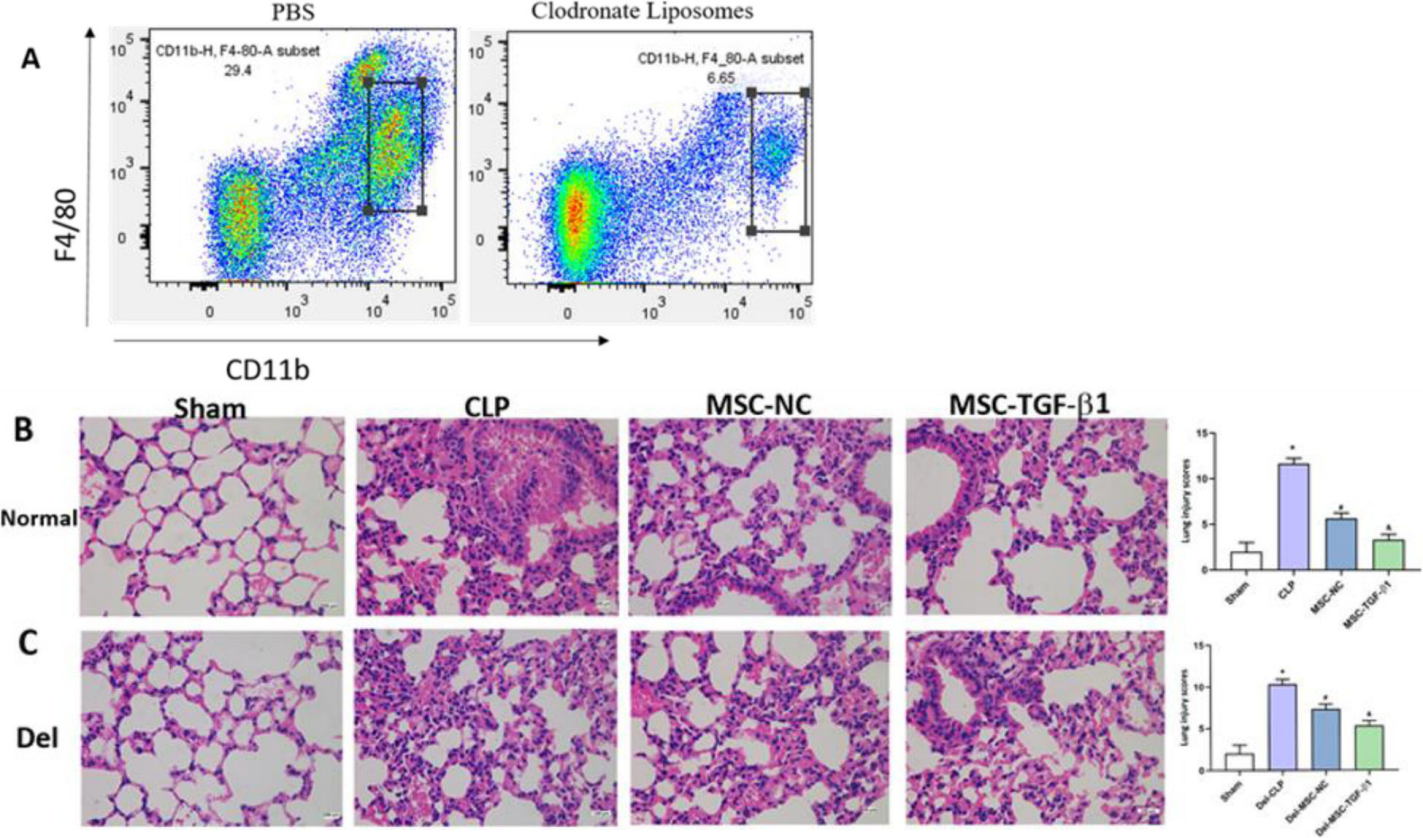
Macrophage depletion weakened the therapeutic effect of MSC-TGF-β1 in septic mice. **a** Depletion of macrophages using clodronate liposomes. **b**, **c** Histopathological images of lung tissues in normal or macrophages-depleted septic mice were obtained by H&E (× 400). Scale bar = 100 μm (*n* = 3, *p < 0.05 vs. the sham group; ^#^*p* < 0.05vs. the CLP group; ^&^*p* < 0.05vs. the MSC-NC group). Del, macrophage depletion; MSC-NC, mesenchymal stem cell carrying GFP; MSC-TGF-β1, mesenchymal stem cell carrying transforming growth factor-beta 1

In order to investigate the important role of macrophages for the therapeutic effects of MSC-TGF-β1 in vivo, mice were depleted of their macrophages by intraperitoneal injection of clodronate liposomes (CL) before MSC-TGF-β1 injection. We first examined the removal efficiency of macrophages in mice (Fig. 7a). Then, we compared the effects of MSC-TGF-β1 in normal and macrophage-depleted septic mice. Our results showed that MSC-TGF-β1 infusion could significantly improve the lung injury in septic mice, while the therapeutic effect of MSC-TGF-β1 was weakened in macrophages-depleted septic mice (Fig. 7b, c). These data demonstrated that macrophages are important cellular mediators of MSC-TGF-β1 therapeutic effects in septic mice.

## Discussion

In recent decades, studies have shown that MSCs are an emerging therapeutic strategy for sepsis and related organ dysfunction due to their unique immunoregulatory properties. MSC infusion can significantly improve the overall survival of septic mice, reducing the level of organ injury [28, 29]. In this study, our data demonstrated that MSC-TGF-β1 exerted a better beneficial effect on CLP-induced septic mice. Intravenous infusion of MSC-TGF-β1 through the tail vein alleviated the histopathological damage of liver, lung, and spleen and reduced the inflammatory response in septic mice. In addition, MSC-TGF-β1 reduced macrophage infiltration in organ and induced macrophage phenotype transformation. Furthermore, the adoptive transfer of MSC-TGF-β1 pretreated macrophage also reduced organ damage in septic mice. These results demonstrated that MSC-TGF-β1 may, through regulating macrophage activation, reduce organ injury and hyper-inflammation in septic mice, which may provide new treatment directions for sepsis.

There is growing evidence that MSCs may be effective in treating septic mice by regulating macrophage phenotype switch from pro-inflammatory to pro-resolution [30]. Gene modification of MSCs before intravenous injection to ensure their stable and long-term expression of specific cytokines can significantly enhance the therapeutic effect of MSCs. For example, a study reported that mesenchymal stem cells overexpressing interleukin-10 promoted neuroprotection in acute ischemic stroke animals [31]. Furthermore, to improve the effective mobilization of MSCs at tissue injury sites, CXCR4 overexpression in MSCs was performed and facilitated the treatment of acute lung injury in rats [4]. In addition, Wang et al. found that IL-10-overexpressing MSCs regulated the inflammatory response and improved the survival of endotoxin-induced ALI mice [20]. Therefore, searching for more effective methods to improve the abilities of MSCs to induce macrophage activation and phenotypic transformation could improve the efficacy of MSCs in treating sepsis.

Recently, studies have supported the idea that paracrine-mediated immune regulation is one of the major mechanisms that drives the beneficial effects of MSCs [30]. Our previous research confirmed that the paracrine effects of MSC-derived TGF-β1 regulated macrophage activation and inhibited macrophage-mediated inflammation [20]. Our present study also showed that recombinant TGF-β1 can improve the organ damage in septic mice, while SB-431542 restrained the effects of rTGF-β1 on septic mice. To examine the therapeutic effect of MSC-TGF-β1 on septic mice, we generated TGF-β1-overexpressing MSCs using a lentiviral vector and transplanted MSC-TGF-β1 into CLP-induced septic mice. Compared with the MSC-GFP control group, the infusion of MSC-TGF-β1 reduced the percentage of macrophages in the lung tissue of septic mice, induced macrophage phenotypic transformation and alleviated inflammation. In addition, we found that the therapeutic effect of MSC-TGF-β1 on macrophage-depleted septic mice was weakened. These data confirm the findings of previous studies showing that clodronate-based depletion of alveolar macrophages in vivo undermines the beneficial effects of MSCs on lung injury [32], suggesting that macrophages are cellular mediators of MSC-TGF-β1. These results collectively indicated that MSC-TGF-β1 effectively regulated macrophage infiltration and phenotypic transformation in organ and thus had an improved therapeutic effect in septic mice.

The uncontrolled inflammatory response and subsequent tissue damage play important roles in the development of sepsis. The paracrine effects of MSC-derived TGF-β1 regulated the phenotypic transformation of macrophages and reduced the macrophage-mediated inflammatory response. Our results showed that MSC-TGF-β1 significantly improved histopathological damage in the lung, liver, and spleen and reduced the expression of pro-inflammatory cytokines in the plasma of septic mice. These results indicated that MSC-TGF-β1 effectively inhibited the inflammatory response and improved tissue damage in septic mice. Furthermore, it is currently believed that cell-cell interactions play an important role in tissue repair. A study reported that after acute tissue injury, rapid tissue re-vascularization and wound healing may benefit from tissue-resident macrophages; this group demonstrated macrophage-derived amphiregulin induced TGF-β activation and the differentiation of pericytes into collagen-producing myofibroblasts [33]. Another study reported that when SiO_2_ was used to induce the activation of lung macrophages, these macrophages acted as effector cells that attracted and regulated other cells by secreting and releasing factors and participated in tissue repair [34]. Therefore, further experiments are needed to clarify the important role played by MSC-TGF-β1-macrophage interactions in the repair of acute tissue injury.

An indirect in vitro co-culture system of MSC-TGF-β1 and LPS-stimulated macrophage could intuitively detect the regulatory effects of MSC-TGF-β1 on macrophage phenotypes. Our results showed that MSC-TGF-β1 effectively induced phenotypic transformation in LPS-stimulated macrophage. Moreover, MSC-TGF-β1 significantly inhibited the expression of IL-6 and IL-1β and promoted the expression of IL-10 in macrophages. These results confirmed at the molecular level that MSC-TGF-β1 could be effective in promoting macrophage phenotypic transformation and reducing macrophage-mediated excessive inflammation.

In our study, we injected macrophages that had been pretreated with MSC-TGF-β1 into septic mice through the tail vein. Our results showed that MSC-TGF-β1 pretreated macrophages significantly improved histopathological damage in the lung, liver, and spleen of septic mice compared to that of mice in the MSC-GFP-pretreated group. This is a new finding regarding the efficacy of macrophage adoptive transfer therapy for CLP-induced sepsis. In addition, several studies reported that adoptive transfer of macrophages also yielded satisfactory results in ARDS, colitis, and airway hyperresponsiveness [35–37]. However, further research is needed to determine the feasibility of these treatments.

This study has several limitations. First, sepsis models of different severities can be generated with CLP. The severe model used in our study and does not represent other severely septic mouse models. Second, TGF-β participates in fibrogenesis as a profibrotic mediator, regulating cell proliferation, apoptosis, migration, and collagen production of fibroblasts [38]. Hence, excessive and/or prolonged TGF-β1 production may have adverse effects, such as promoting the development of pulmonary fibrosis. Therefore, we need to further explore the therapeutic effect of MSC-TGF-β1 on septic mice in the further.

## Conclusion

In summary, our findings show that, in the inflammatory environment of sepsis, TGF-β1 overexpression enhances the effect of MSCs on organ injury in CLP-induced septic mice. MSC-TGF-β1 modulates the infiltration of macrophages in the organ of septic mice, promotes the transformation of CD86^+^ macrophages to the CD206^+^ phenotype, and reduces the production of pro-inflammatory cytokines. Moreover, our study suggests that the infusion of macrophages treated with MSC-TGF-β1 exerts protective effects against organ injury in vivo. This study provides a new strategy for improving MSC-based treatments for sepsis.

## Supplementary information

**Additional file 1: Figure S1.** The therapeutical effects of rTGF-β1 on organs injury in septic mice. The tissue sections were stained with haematoxylin–eosin. (a, b, c): Histopathological images of lung, liver and spleen tissues (H&E staining, 400×). Scale bar = 20 μm. (d, e,f, ): The injury scores of lung、 liver and spleen. (*n*=3; *p<0.05 vs. sham group; ^#^p<0.05 vs. CLP group; ^&^p<0.05 vs. rTGF-β1 group). Abbreviations: CLP: cecal ligation and puncture; rTGF-β1, recombinant TGF-β1;SB, SB-431542. **Figure S2.** The body temperature in different groups. (*n*=3; *p<0.05 vs. sham group; ^#^p<0.05 vs. CLP group). Abbreviations: MSCs: Mesenchymal stem cell; MSC-NC: MSC normal control; MSC-TGF-β1: TGF-β1 overexpressing MSCs. **Figure S3.** The distribution of MSC-TGF-β1 in septic mice after intravenous infusion at 24 hours. Fluorescence microscopy validation in recipient lung、liver and spleen. MSC-TGF-β1 (green) were observed in the lung、liver and spleen tissues. Nuclei were stained with DAPI (blue). Scale bar=20 μm. Abbreviations: CLP: cecal ligation and puncture; MSC-TGF-β1: TGF-β1 overexpressing MSCs. **Figure S4.** The level of TGF-β1 in the plasma were tested by ELISA (n = 3, *p<0.05 vs. sham group; ^#^p<0.05 vs. CLP group; &*p* < .05 vs. MSC-NC group). MSCs: Mesenchymal stem cell; MSC-NC: MSC normal control; MSC-TGF-β1: TGF-β1 overexpressing MSCs. **Figure S5.** The concentration of inflammatory cytokines in the supernatant of MSC medium. MSCs: Mesenchymal stem cell; MSC-NC: MSC normal control; MSC-TGF-β1: TGF-β1 overexpressing MSCs. **Figure S6.** Effect of primary peritoneal macrophages treated with MSC-TGF-β1 on organ injury in CLP-induced septic mice. (a): Histopathological images of lung tissues were obtained by H&E (400×). Scale bar=100 μm. (b): Injury scores for the lung from each group. (n = 3, *p < .05 vs. the sham group; #p < .05 vs. the LPS group; &p<0.05 vs. the MSC-Ma group. Abbreviations: Abbreviations: Ma, macrophages; MSCs, mesenchymal stem cell; MSC-Ma, MSCs preconditioned Macrophage; MSC-NC-Ma, MSC-NC preconditioned Macrophage; MSC-TGF-β-Ma, MSC-TGF-β preconditioned macrophage; MSC-NC: mesenchymal stem cell carrying GFP; MSC-TGF-β1: TGF-β1 overexpressing MSC. **Figure S7.** The identification of MSCs. (A) MSCs surface markers, including CD29, CD44, CD117, Sca-1and CD31 were determined by flow cytometry. (B) The morphology of MSC at the 6th passage (×100) and the multilineage differentiation capacities of MSC, including adipogenic differentiation staining with oil red-O (C, ×200), osteogenic differentiation staining with alizarin red (D, ×200) were observed with a microscope. (Data were provided by Cyagen Bioscience, Inc., Guangzhou, China).

## Data Availability

All data generated or analyzed during this study are included in this published article.

## Abbreviations

CLP: Cecal ligation and puncture
Del: Macrophage depletion mice
DMEM/F12: Dulbecco’s modified Eagle’s medium/F12
ELISA: Enzyme-linked immunosorbent assay
GFP: Fluorescent protein
H&E: Hematoxylin-eosin
MSCs: Mesenchymal stem cells
MSC-Ma: MSCs preconditioned macrophage
MSC-NC: Mesenchymal stem cells carrying green fluorescent protein (normal control)
MSC-NC-Ma: MSC-NC preconditioned macrophage
MSC-TGF-β1: MSCs overexpressing TGF-β1
TGF-β1: Transforming growth factor-beta 1
MSC-TGF-β1-Ma: MSC-TGF-β1 preconditioned macrophage
