# Correction: Overexpressing TGF-β1 in mesenchymal stem cells attenuates organ dysfunction during CLP-induced septic mice by reducing macrophage-driven

**DOI:** 10.1186/s13287-022-03078-6

**Published:** 2022-07-27

**Authors:** Feng Liu, Jianfeng Xie, Xiwen Zhang, Zongsheng Wu, Shi Zhang, Ming Xue, Jianxiao Chen, Yi Yang, Haibo Qiu

**Affiliations:** Department of Critical Care Medicine, School of Medicine, Zhongda Hospital, Southeast University, Nanjing, 210009 China

Correction to: Stem Cell Research & Therapy (2020) 11:378 https://doi.org/10.1186/s13287-020-01894-2

The original article [1] contains a number of errors which the authors would like to clarify:

1. Following publication of the original article [1], the authors identified an incorrect image in Fig. 3. Correction made is listed below.
  Fig. 3F: Spleen: MSC-TGF-β1 was misplaced, and now corrected

**Fig. 3:**
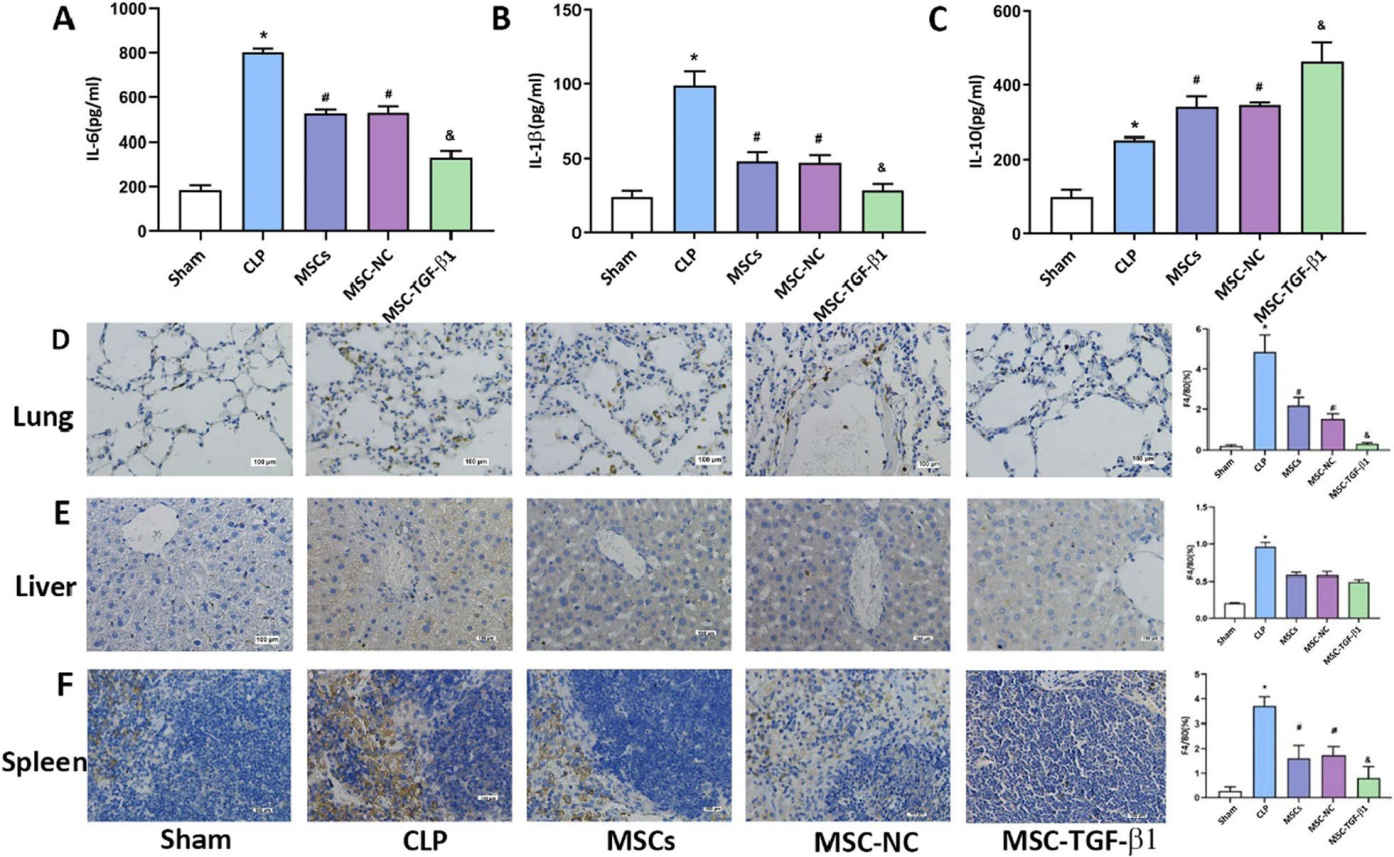
Effect of MSC-TGF-β1 on inflammatory cytokines in the plasma and macrophages in tissue. **a**, **b**, **c**: The levels of IL-6, IL-1β, and IL-10 in the plasma were tested by ELISA (n = 3, *p < 0.05 vs. the sham group; #*p* < 0.05 vs. the CLP group; &*p* < 0.05 vs. the MSC-NC group). **d**, **e**, **f**: The levels of macrophages in lung, liver and spleen tissue were assayed by immumohistochemical staining. (n = 3, *p < 0.05 vs. the sham group; #*p* < 0.05 vs. the CLP group; &*p* < 0.05 vs. the MSC-NC group). Scale bar = 100 μm. Abbreviations: CLP: cecal ligation and puncture; MSCs, mesenchymal stem cells; MSC-NC: mesenchymal stem cell carrying GFP; MSC-TGF-β1: TGF-β1 overexpressing MSC

(2) In the Materials and Methods section under the sub-heading “**CLP model of sepsis**”, the term, ‘5% chloral hydrate (400 mg/kg)’ should be pentobarbital (50 mg/kg), this can be seen in the first draft submitted to the journal. Correction made is listed below.
  The sepsis model was induced via the CLP method. Briefly, the mice were anaesthetized with pentobarbital (50 mg/kg) by intraperitoneal injection, and their lower abdomen was then shaved.
(3) In the section of ethics approval and consent to participate: All of the experimental procedures were approved by the Southeast University Ethics Committee (protocol number: 20171101006).

These corrections will not affect the result and scientific conclusion of the manuscript. The authors would like to apologize for any inconvenience caused.
