# Supplementary material for: Overexpressing TGF-β1 in mesenchymal stem cells attenuates organ dysfunction during CLP-induced septic mice by reducing macrophage-driven inflammation
Source: Stem Cell Res Ther. 2020 Sep 3;11:378. doi: 10.1186/s13287-020-01894-2 (PMC7469348; doi:10.1186/s13287-020-01894-2)
Supplement: Supplementary file 1 — Additional file 1: Figure S1. The therapeutical effects of rTGF-β1 on organs injury in septic mice. The tissue sections were stained with haematoxylin–eosin. (a, b, c): Histopathological images of lung, liver and spleen tissues (H&E staining, 400×). Scale bar = 20 μm. (d, e,f, ): The injury scores of lung、 liver and spleen. (n=3; *p<0.05 vs. sham group; #p<0.05 vs. CLP group; &p<0.05 vs. rTGF-β1 group). Abbreviations: CLP: cecal ligation and puncture; rTGF-β1, recombinant TGF-β1;SB, SB-431542. Figure S2. The body temperature in different groups. (n=3; *p<0.05 vs. sham group; #p<0.05 vs. CLP group). Abbreviations: MSCs: Mesenchymal stem cell; MSC-NC: MSC normal control; MSC-TGF-β1: TGF-β1 overexpressing MSCs. Figure S3. The distribution of MSC-TGF-β1 in septic mice after intravenous infusion at 24 hours. Fluorescence microscopy validation in recipient lung、liver and spleen. MSC-TGF-β1 (green) were observed in the lung、liver and spleen tissues. Nuclei were stained with DAPI (blue). Scale bar=20 μm. Abbreviations: CLP: cecal ligation and puncture; MSC-TGF-β1: TGF-β1 overexpressing MSCs. Figure S4. The level of TGF-β1 in the plasma were tested by ELISA (n = 3, *p<0.05 vs. sham group; #p<0.05 vs. CLP group; &p < .05 vs. MSC-NC group). MSCs: Mesenchymal stem cell; MSC-NC: MSC normal control; MSC-TGF-β1: TGF-β1 overexpressing MSCs. Figure S5. The concentration of inflammatory cytokines in the supernatant of MSC medium. MSCs: Mesenchymal stem cell; MSC-NC: MSC normal control; MSC-TGF-β1: TGF-β1 overexpressing MSCs. Figure S6. Effect of primary peritoneal macrophages treated with MSC-TGF-β1 on organ injury in CLP-induced septic mice. (a): Histopathological images of lung tissues were obtained by H&E (400×). Scale bar=100 μm. (b): Injury scores for the lung from each group. (n = 3, *p < .05 vs. the sham group; #p < .05 vs. the LPS group; &p<0.05 vs. the MSC-Ma group. Abbreviations: Abbreviations: Ma, macrophages; MSCs, mesenchymal stem cell; MSC-Ma, MSCs preconditioned Macrop [file 13287_2020_1894_MOESM1_ESM.docx]

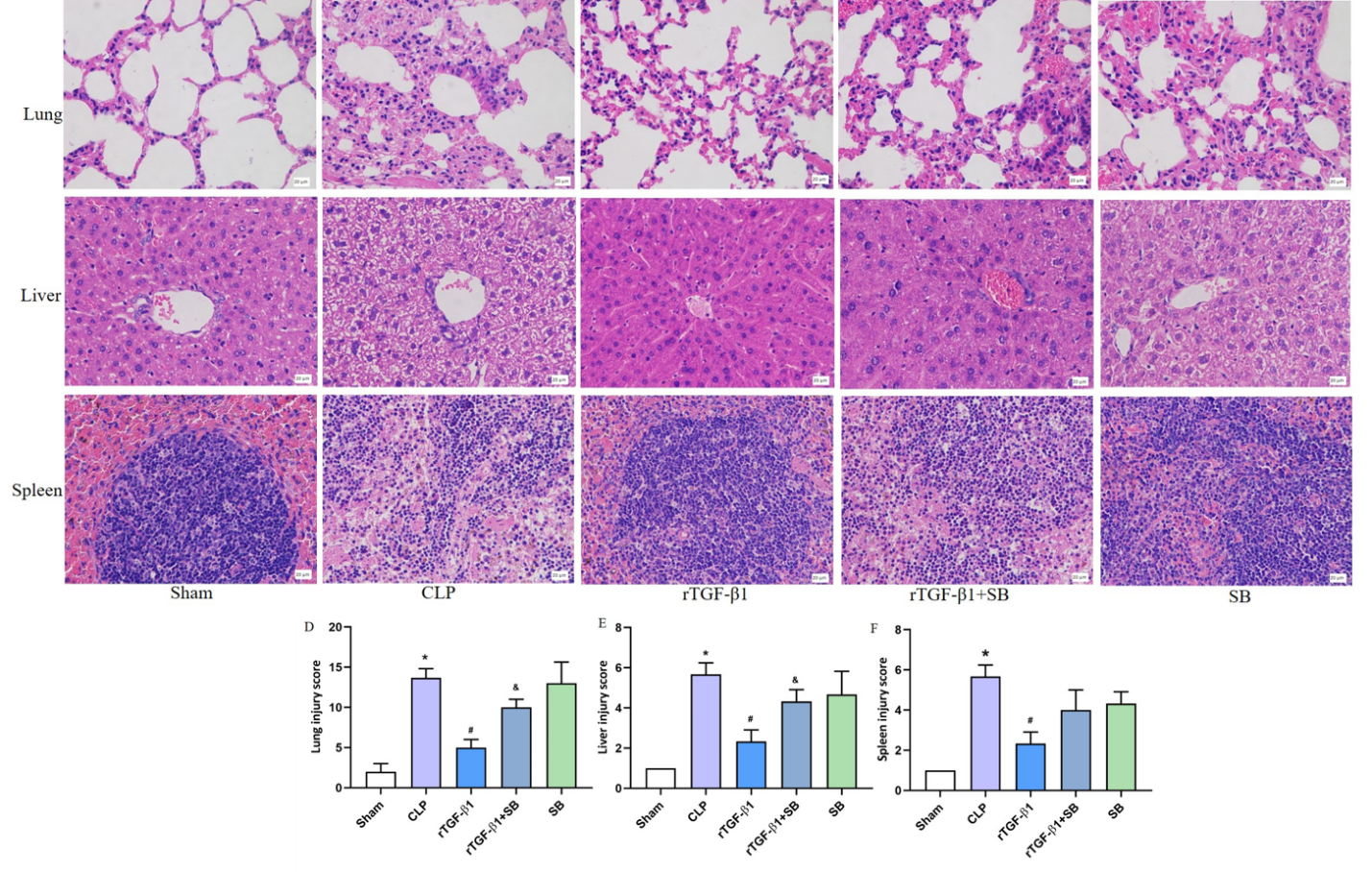


**Fig. S1**. The [therapeutical](javascript:;) [effect](javascript:;)s of rTGF-β1 on organs injury in septic mice. The tissue sections were stained with haematoxylin–eosin. (a, b, c): Histopathological images of lung, liver and spleen tissues (H&E staining, 400×). Scale bar = 20 μm. (d, e,f, ): The injury scores of lung、 liver and spleen. (n=3; *p<0.05 vs. sham group; ^#^p<0.05 vs. CLP group; ^&^p<0.05 vs. rTGF-β1 group). Abbreviations: CLP: cecal ligation and puncture; rTGF-β1, recombinant TGF-β1；SB, SB-431542.


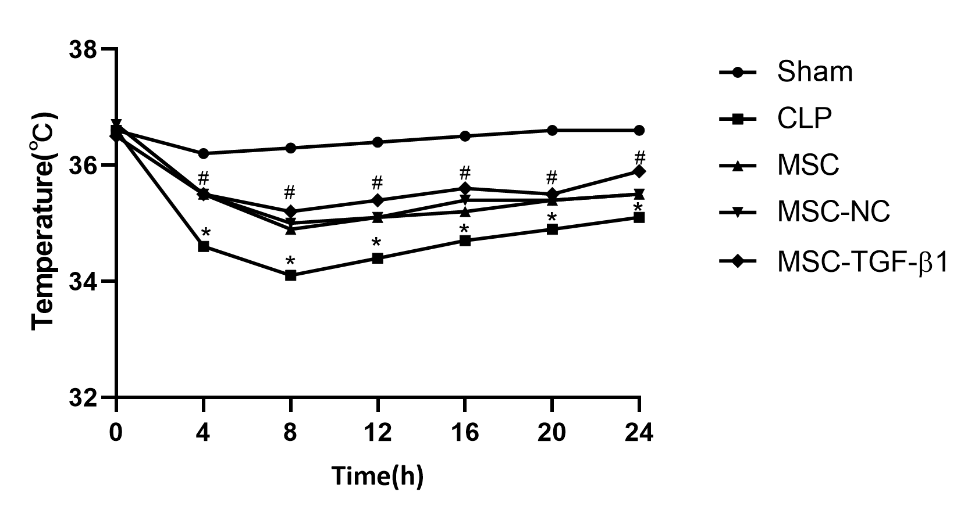


**Fig. S2.** The body temperature in different groups. (n=3; *p<0.05 vs. sham group; ^#^p<0.05 vs. CLP group). Abbreviations: MSCs: Mesenchymal stem cell; MSC-NC: MSC normal control; MSC-TGF-β1: TGF-β1 overexpressing MSCs.


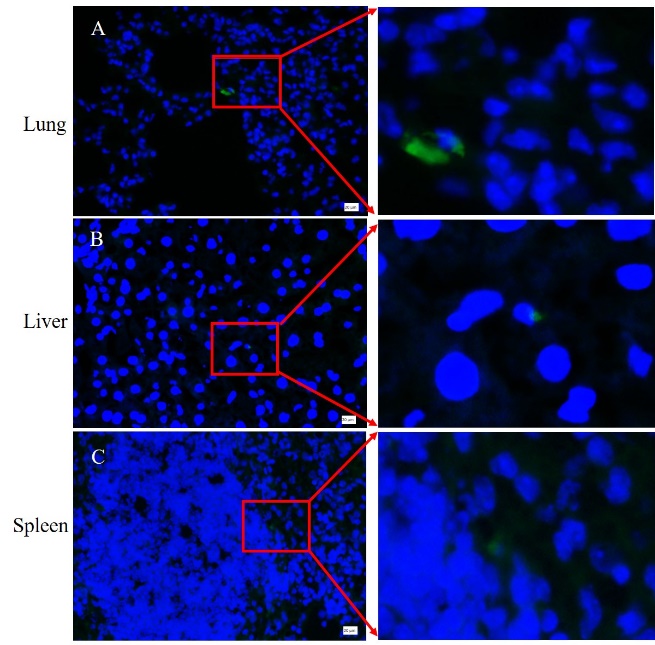


**Fig. S3.** The distribution of MSC-TGF-β1 in septic mice after intravenous infusion at 24 hours. Fluorescence microscopy validation in recipient lung、liver and spleen. MSC-TGF-β1 (green) were observed in the lung、liver and spleen tissues. Nuclei were stained with DAPI (blue). Scale bar=20 μm. Abbreviations: CLP: cecal ligation and puncture; MSC-TGF-β1: TGF-β1 overexpressing MSCs.


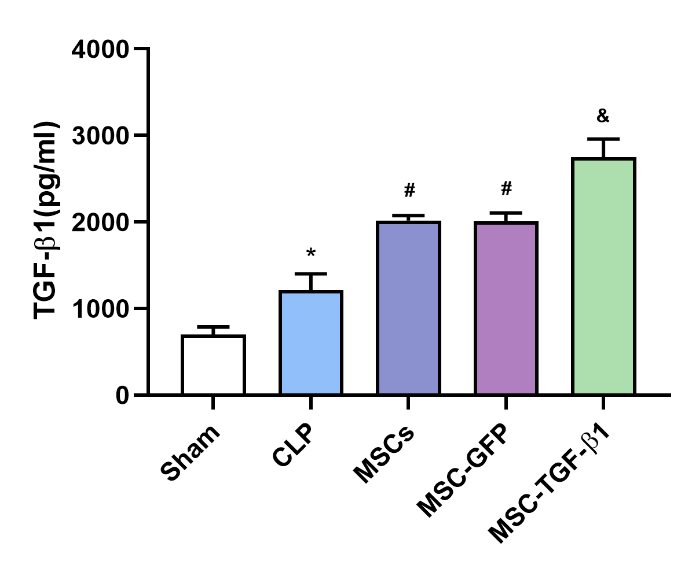


**Fig. S4**.The level of TGF-β1 in the plasma were tested by ELISA (n = 3, *p<0.05 vs. sham group; ^#^p<0.05 vs. CLP group; &p < .05 vs. MSC-NC group). MSCs: Mesenchymal stem cell; MSC-NC: MSC normal control; MSC-TGF-β1: TGF-β1 overexpressing MSCs.


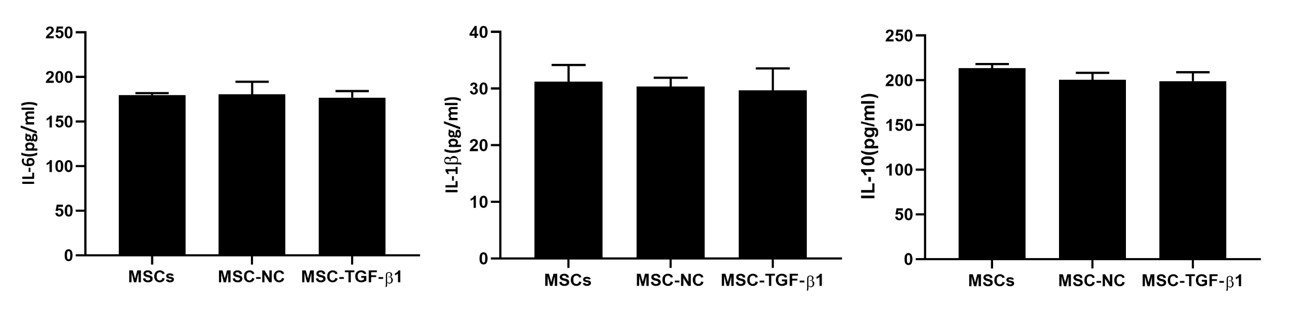


**Fig. S5.** The concentration of inflammatory cytokines in the supernatant of MSC medium. MSCs: Mesenchymal stem cell; MSC-NC: MSC normal control; MSC-TGF-β1: TGF-β1 overexpressing MSCs.

**
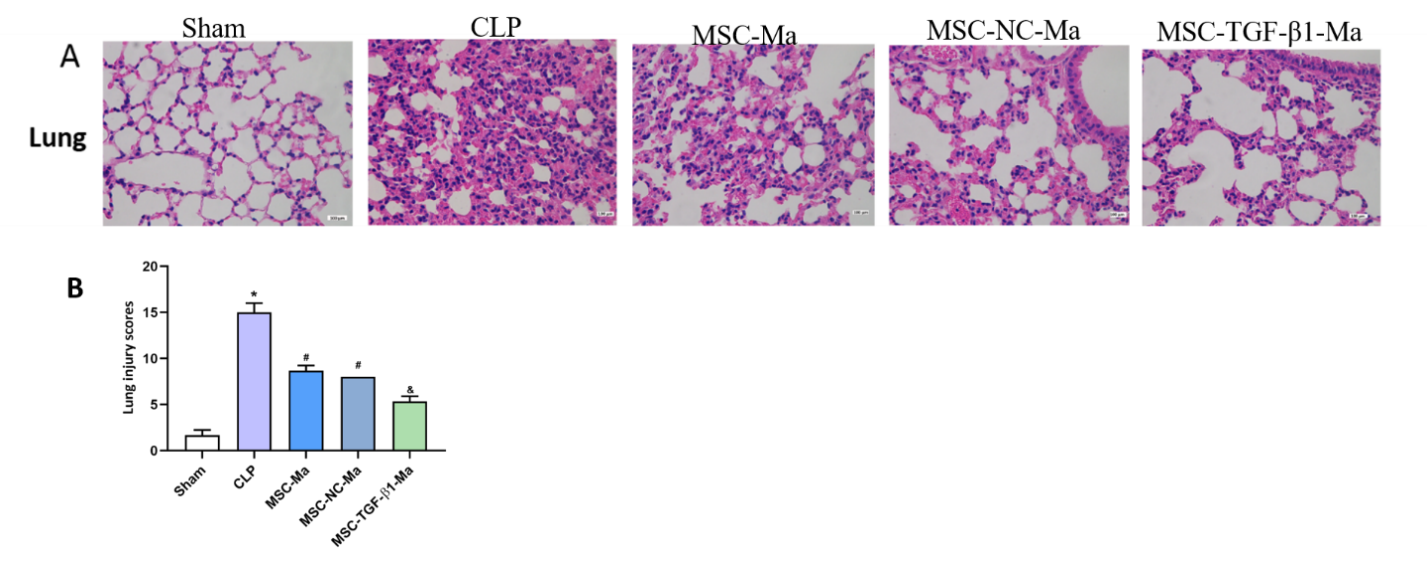
**

**Fig. S6.** Effect of primary peritoneal macrophages treated with MSC-TGF-β1 on organ injury in CLP-induced septic mice. (a): Histopathological images of lung tissues were obtained by H&E (400×). Scale bar=100 μm. (b): Injury scores for the lung from each group. (n = 3, *p < .05 vs. the sham group; #p < .05 vs. the LPS group; &p<0.05 vs. the MSC-Ma group. Abbreviations: Abbreviations: Ma, macrophages; MSCs, mesenchymal stem cell; MSC-Ma, MSCs preconditioned Macrophage; MSC-NC-Ma, MSC-NC preconditioned Macrophage; MSC-TGF-β-Ma, MSC-TGF-β preconditioned macrophage; MSC-NC: mesenchymal stem cell carrying GFP; MSC-TGF-β1: TGF-β1 overexpressing MSC.


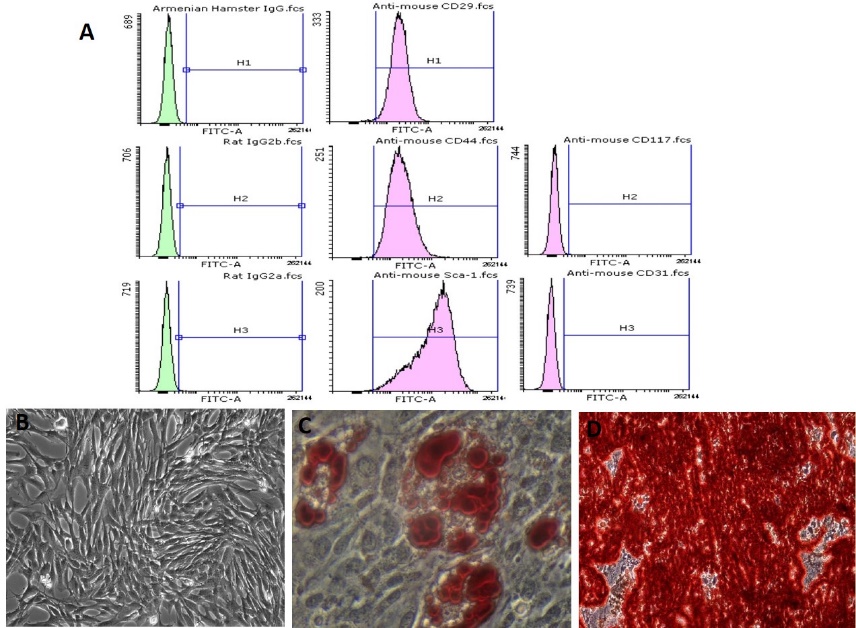


**Fig. S7.** The identification of MSCs. (A) MSCs surface markers, including CD29, CD44, CD117, Sca-1and CD31 were determined by flow cytometry. (B) The morphology of MSC at the 6^th^ passage (×100) and the multilineage differentiation capacities of MSC, including adipogenic differentiation staining with oil red-O (C, ×200), osteogenic differentiation staining with alizarin red (D, ×200) were observed with a microscope. （Data were provided by Cyagen Bioscience, Inc., Guangzhou, China）
